# The role of bile acids in nutritional support

**DOI:** 10.1186/s13054-018-2160-4

**Published:** 2018-09-30

**Authors:** Gustav van Niekerk, Tanja Davis, Willem de Villiers, Anna-Mart Engelbrecht

**Affiliations:** Stellenbosch University, Stellenbosch Central, Stellenbosch, 7599 South Africa

**Keywords:** Bile acids, Autophagy, Farnesoid-X-receptor, Inflammation, Nutritional support, Sepsis

## Abstract

Nutritional support continues to receive much attention as a possible intervention to prevent loss of lean tissue mass, promote recovery and re-establish proper immune function in critical care patients. Yet there remains much controversy regarding the clinical efficacy of such interventions. In addition to the direct effect of nutrition in terms of micro- and macronutrient content, nutritional formulations may exert an effect via the physiological response to feeding. Here, we highlight the key role of postprandial reabsorbed bile acids in attenuating both the inflammatory response and autophagy. These observations suggest that not all patients would benefit from aggressive nutritional support.

## Introduction

Three observations strongly suggest that sickness-associated anorexia (SAA) serves an important function during an infection. First, the fact that inflammatory mediators such as interleukin-1 beta (IL-1β), IL-6 and tumour necrosis factor (TNF) induce SAA [1] suggests that SAA is a dedicated response enacted by the host during an infection or severe trauma and not a manifestation of pathology per se. Second, the observation that a loss of appetite manifests in model organisms as well as in domestic and companion animals strongly suggests that SAA represents an evolutionarily conserved response, indicating that selective pressure has maintained this response on the basis of its functional value. Thus, from an evolutionary perspective, these considerations suggest that avoiding food intake likely plays an important adaptive function during an inflammatory response such as is invariably witnessed in critical care patients. Finally, it is worth noting that inflammatory mediators antagonise the digestive process. Cytokines such as IL-1β, IL-6 and TNF-α, through various mechanisms, decrease bile flow (reviewed in [2]). Also, TNF suppresses gastric motility through its action on the dorsal vagal complex [3]. This illustrates that inflammatory mediators enact a range of physiological responses intended to limit the intake of food, strongly supporting the notion that a decrease in feeding represents a dedicated response to inflammatory mediators.

Notwithstanding these considerations, nutritional support is administered in various contexts. The implementation of aggressive nutritional support is motivated by two considerations. First, it is often argued that a decrease in appetite removes the incentive for foraging behaviour, thereby conserving energy for other critical functions such as the mobilisation of the immune response. However, nutritional support can be administered to patients without the associated metabolic cost of gathering food. Second, activation of an inflammatory response is associated with a remarkable increase in metabolic rate, leading to a severe nitrogen deficit and the loss of lean tissue mass. In addition, critically ill patients are most often immobile which likely aggravates the catabolism of lean tissue. Since the loss of lean mass is associated with an increase in mortality and morbidity, nutritional support not only may halt the extensive catabolism in critically ill patients but also may avert malnutrition. These considerations then provide a rational justification for implementing nutritional support in critically ill patients.

Despite these considerations, there is currently no single study providing “unequivocal evidence that feeding protocols targeting full-replacement nutrition early in the course of critical illness result in clinical benefits” [4]. A number of factors have been pointed out to contribute to the controversy, including variation in the “timing, route, and composition of nutritional interventions” [5]. Another aspect seldom addressed is that nutritional support likely exerts its effect not only through the nutritional composition of nutrients (micro- and macronutrients) but also through the physiological response to food.

Here, we argue that a major mechanism by which nutritional support mediates its therapeutic effects relates to the hormonal effect of postprandial reabsorbed bile acids (BAs). In particular, BAs may attenuate the inflammatory response transcriptionally but also could compromise lipopolysaccharide (LPS) clearance by attenuating the expression of scavenger receptors as well as autophagy, a cell survival response. Emerging evidence has also implicated BAs as transcriptional inhibitors of autophagy. Furthermore, evidence has demonstrated that BAs alter the vascular system, suggesting that postprandial reabsorption of BAs may have an impact on the development of shock. Finally, we briefly point out that nutritional support may exert other clinically relevant effects such as altering the microbiome. Taken together, these observations strongly suggest that, during an infection, nutritional support may curtail immunological functions and compromise autophagy but conversely may also prove beneficial in averting an excessive inflammatory response to sterile injury.

## Bile acids: Modulating inflammation

Following a meal, fatty acids in the lumen of the gut induce the release of cholecystokinin, which in turn causes the sphincter of Oddi to relax and bile to flow into the small intestine. Of note, the fatty acid chain length plays a vital role in regulating the release of cholecystokinin, and long-chain fatty acids provide a much more pronounced release of cholecystokinin [6, 7]. Mechanistically, long-chain fatty acids activate the G protein−coupled receptor 40 (GPR40) of cells in the small intestine, promoting the release of cholecystokinin [8]. Thus, fatty acid composition of enteral nutrition directly influences BA release. In the intestine, BAs are de-conjugated (resulting in secondary BAs) or modified in various other ways by intestinal microbiota. Following a meal, most of these BAs are reabsorbed: despite a large capacity for bile synthesis, about 95% of BAs are reabsorbed both actively and passively, entering the mesenteric vein which drains into the hepatic portal vein before finally being absorbed by liver cells in the sinusoidal space of the liver [9–11].

Although BAs are typically associated with digestion, BAs have long been known to mediate an anti-inflammatory effect. As an example, BAs induced a 50% suppression of IL-6, IL-1 and TNF-α in monocytes at a concentration of between 60 and 80 μmol/L [12]. Similarly, in earlier studies, it was reported that at a concentration of 120 μM, the secondary BA deoxycholic acid suppressed the expression of TNF-α by 90% but only slightly (10%) suppressed TNF-α expression at 25 μM [13]. Even though these observations demonstrate anti-inflammatory effects of BAs, it also is apparent that the effects of BAs are highly dose-dependent. Thus, it should be noted that many studies employed high levels of BAs that may not always be applicable to all organ systems. Normal fasting serum BA concentration is usually below 2.5 μM and may raise to a postprandial maximum three times above the fasting level. However, BA concentrations are much higher within the hepatic portal system. Fasting BA concentrations in the venous portal system averaged 14 μM (compared with the 2.4 μM in the peripheral venous system) and exhibit a postprandial peak of 43 μM [14], which is closer to the physiologically effective dosage range at which inflammation is attenuated. This suggests that reabsorbed BAs may primarily exert their greatest effect on the liver.

Mechanistically, it is now well established that the reabsorbed secondary BAs act as hormones targeting cell surface G protein–coupled BA receptors (such as Takeda G protein–coupled receptor 5, or TGR5) and the nuclear receptor, farnesoid-X-receptor (FXR) [15], which also play a role in modulating the immune system. As an example, activation of TGR5 by BAs suppressed the of inflammatory cytokines by LPS-challenged Kupffer cells [16]. Correspondingly, FXR activation attenuates nuclear factor-kappa B (NF-κB) gene transcription induced by IL-1β in vascular smooth muscle cells [17]. FXR activation also resulted in a significant suppression of interferon-gamma (IFNγ)-responsive genes in macrophages [18]. It is thus evident that activation of FXR and TGR5 by BAs is capable of attenuating an inflammatory response.

BAs may also indirectly alter the inflammatory response by compromising LPS clearance. The scavenger receptor class B type I (SR-BI) plays an important role in transporting cholesterol associated with high-density lipoprotein (HDL) into a range of cells, including macrophages and hepatocytes [19]. LPS also associates with HDL, which suggests that LPS may be cleared by the uptake of HDL through SR-BI. Indeed, this line of reasoning is supported by findings from Cai et al., who demonstrated that mice lacking SR-BI exhibited decreased clearance of LPS and an accompanied increase in the inflammatory response to an LPS challenge [20], highlighting the role of SR-BI in clearing LPS. Yet it is also known that BAs, via their function on FXR, suppress the hepatic expression of SR-BI [21]. Thus, the postprandial reabsorption of BAs may reduce SR-BI expression, thereby compromising the rate by which LPS is internalised and subsequently cleared form circulation.

Taken together, these observations demonstrate that nutritional support may exert clinically relevant effects through the subsequent reabsorption of BAs. The effect of BAs may be particularly relevant in the context of bacterial infection: given the observation that hepatic clearance of LPS is important in preventing excessive inflammation [22], it is possible that nutritional support results in elevated inflammation by compromising the expression of SR-BI and the subsequent clearance of LPS. Simultaneously, the anti-inflammatory effect of BAs may result in a compromised immune response. In this regard, it is worth noting that sepsis is often characterised by a robust inflammatory response coupled with an immune-suppressive state [23, 24]. The extent to which the reabsorption of BAs may contribute towards such immune dysfunction thus needs to be evaluated.

## Bile acids: Modulating autophagy

Another important consequence of feeding is the suppression of autophagy. Here again, the postprandial reabsorption of BAs has been shown to attenuate autophagy transcriptionally via the activation of FXR. FXR activation suppresses the expression of various proteins involved in autophagy by antagonising the function of other transcription factors such as cAMP response element-binding protein (CREB) [25]. Similarly, Lee *et al*. [26] recently demonstrated that FXR competes with peroxisome proliferator–activated receptor-alpha (PPARα) for binding sites in the promoter regions of various autophagic proteins with different transcriptional outcomes: whereas PPARα induces the expression of autophagic proteins, FXR dramatically suppresses autophagy. Thus, by antagonising the function of other transcription factors such as CREB and PPARα, FXR plays a prominent role as a regulator of autophagy during feed-fasting cycles. This also suggests that enteral nutrition may lead to the suppression of autophagy as a result of FXR activation by reabsorbed BAs, consistent with previous reports suggesting that autophagy may be attenuated by the administration of nutritional support in rabbits [27] and possibly also in humans [28].

Indirect evidence also suggests that the inhibition of autophagic/lysosomal function may result in a compromised ability to clear LPS during an infection. Earlier studies have documented the localisation of LPS within lysosomal vesicles [29]. In this regard, lysosomal vesicles contain the enzyme acyloxyacyl hydrolase (AOAH) [30], which is known to detoxify LPS [31], suggesting that the autophagic machinery may play an important role in the detoxification of LPS. This may be particularly important during an infection when bile flow is low since most LPS is usually removed through bile secretion [32]. In support of this view is that mice lacking the key autophagic protein, Atg16L1, exhibit enhanced sensitivity to LPS and an increased release of IL-1β and IL-18 [33]. Similarly, in other preclinical studies using rodent models, autophagy induction was shown to be protective in a range of organs during sepsis [34–36], including the liver [37, 38]. It is therefore likely that BAs curtail the clearance of LPS, both by transcriptionally inactivating SR-BI expression [21] and by preventing lysosomal detoxification of LPS.

## Bile acids as mediators of nutritional support

It is thus evident that increased BA reabsorption as a result of nutritional support may alter the immune response and impact on the inflammatory status of a patient. However, BAs have also been implicated in a range of other clinically relevant contexts. As an nutritional support may also complicate haemodynamics, not only by directly increasing blood flow towards intestines but also indirectly through the effect of BAs on the liver. It has long been noted that BAs cause an increase in blood flow through the splanchnic circulation [39]. In hepatic sinusoidal endothelial cells isolated from rats, BA (25 μM taurolithocholate) activates TGR5, resulting in increased expression of endothelial nitric oxide (NO) synthase [40]. Given the large blood volume of the liver, the increase in NO may lead to an increase in blood vessel dilatation, suggesting that BAs may contribute to a decrease in blood pressure.

There is also a need to evaluate the role of BAs in other peripheral systems beyond the liver. BAs have been shown to alter haemodynamic parameters outside of the enterohepatic circulation, which collectively result in decreased blood pressure (reviewed in [41]). This includes effects on the heart, as is evident by the observation that BAs can decrease contractility of cardiomyocytes [42], and also the vasculature, as BAs induce vasodilatation [43]. However, most of these studies have been conducted in the context of cholestasis or with levels of BAs elevated above the normal postprandial levels. Consequently, it is not to what extent nutritional support may induce similar effects.

Other evidence suggests that BAs may have a protective role in liver regeneration. Cholic acid has been shown to promote liver regeneration in mice in an FXR-dependent manner [44]. Similar findings were reported by others, demonstrating that physiologically relevant levels of BAs can promote the differentiation of hepatocytes from mesenchymal stem cells derived from either human or rat liver, bone marrow and umbilical cord blood while also suppressing the expression of proteins associated with fibrosis, such as COL1α2 and α-SMA [45]. Collectively, these observations suggest that BAs may promote liver regeneration.

Conversely, in both rats [46] and humans [47, 48], high serum BA levels predict short-term mortality, perhaps suggesting that BAs may have a negative impact on patient outcome. However, both severe inflammation [49] and liver damage [50] are also known to increase serum BA levels, suggesting that an increase merely reflects disease severity rather than a directly contributing factor to pathology. Additionally, primary BAs are modified to secondary BAs in the intestinal tract by microbiota, altering the functions of BAs. As an example, primary (unmodified) BAs, but not secondary (modified) BAs, induce the expression of CXCL16 in hepatic sinusoidal endothelial cells, resulting in increased infiltration by natural killer T cells in the liver [51]. This observation suggests that reabsorbed secondary BAs may have a clinical impact different from that of primary BAs during cholestasis. In summary, the relationship between postprandial BAs and elevated BAs manifesting in other disease states is unclear and warrants further investigation.

**Fig. 1:**
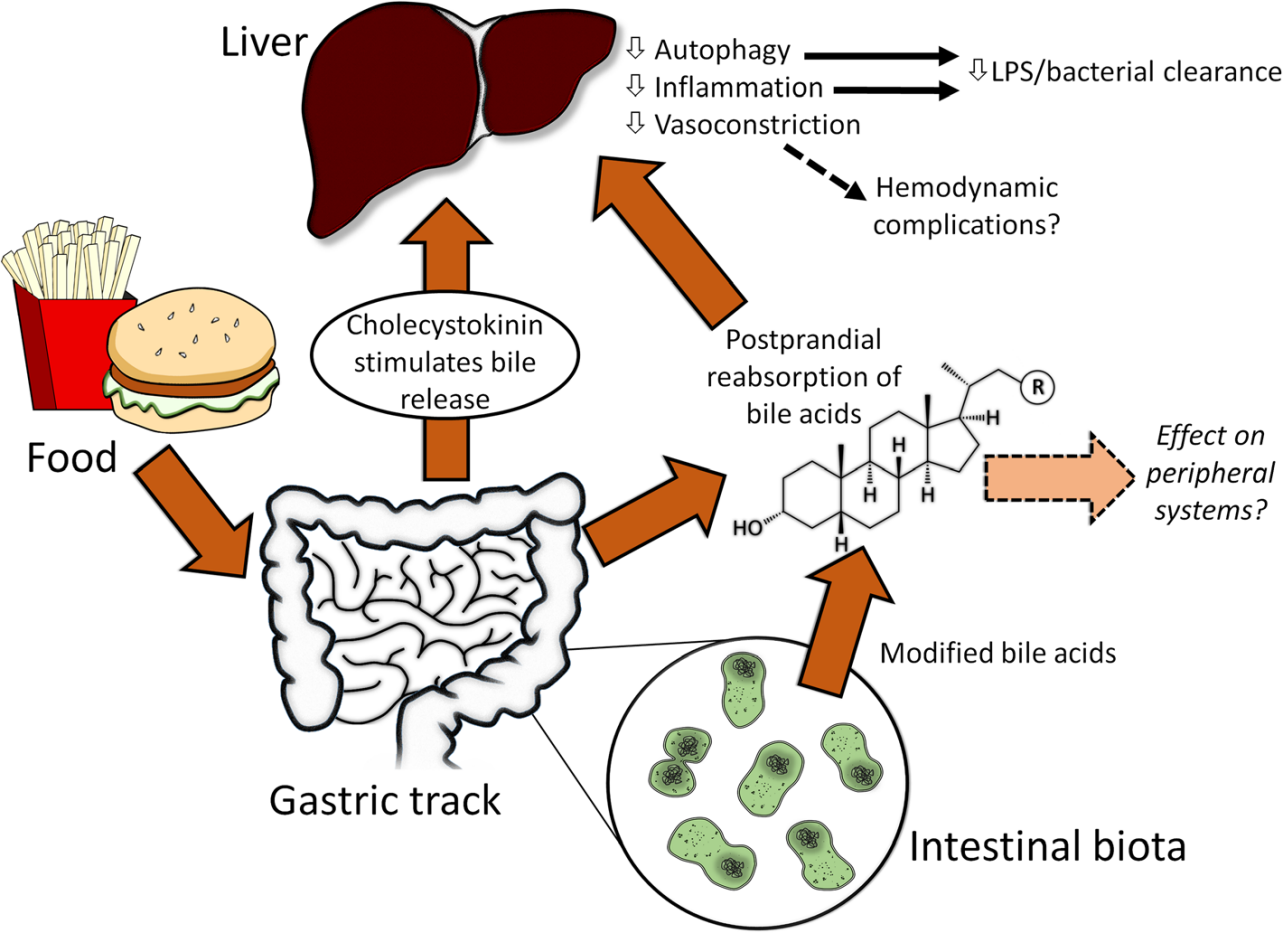
Bile acids (BAs) released in response to food are modified by intestinal microbiota before being reabsorbed. Reabsorbed BAs may exert a number of effects on the liver, including attenuating the inflammatory response, diminishing autophagic activity and inducing nitric oxide–mediated vasodilatation. Since BAs are effectively reabsorbed, systemic levels may be much lower and the effects of BAs may be less pronounced. Abbreviation: *LPS* lipopolysaccharide

Taken together, these observations highlight a number of mechanisms by which BAs mediate clinically relevant physiological response (Fig. 1). It is thus evident that nutritional support, through its effects on the release and subsequent reabsorption of BAs, may benefit some but not all patients. There is thus a clear need to explore the physiological consequence of nutrition, including the downstream effects of BAs.

Finally, although we have focused on postprandial BAs, it is also worth noting that other elements of nutrition may have adverse effects on critically ill patients. As an example, iron is the rate-limiting element in bacterial growth, and it is now well established that iron withdrawal plays an adaptive immunological function directed at starving bacteria of this critical element [52]. Yet it is not unusual to find enteral feeding formulations that are supplemented with iron (for example, ferrous sulphate). Evidence from patients with sepsis also implicates a role for intestinal microbiota in the disease trajectory. Evaluating metabolites in the urine of patients with sepsis revealed an increase in ethanol as a prognostic biomarker for poor outcome [53]. These authors pointed out that, since these metabolites persisted for 24 h after hospital admission, where patients either received a controlled diet or fasted, the ethanol must be derived from fermentation by the microbiota. This raises the possibility that complex starch such as maltodextrin, a major component of enteral nutritional formulas, in fact provides fermentable substrates that increase ethanol production by intestinal microbiota. These observations suggest that nutritional support may have clinically relevant consequences that extend beyond addressing the nutritional need of the patient.

## Conclusion

It is thus clear that elevation of BAs resulting from nutritional support may alter autophagy and inflammation and possibly impact the development of shock. Collectively, these observations suggest that nutritional support may be more beneficial in patients where an immediate threat of infection is not present or where an anti-inflammatory effect is pursued. However, it is not clear how elevated BAs after a meal and the elevation of BAs in other disease states (for example, inflammation-induced cholestasis) are functionally related. There is also a need for more targeted studies aimed at elucidating the direct effect of BAs and the possible therapeutic application of BA supplementation in critically ill patients. Finally, the potential impact of postprandial reabsorbed BAs highlights an important consideration: nutritional support may exert effects that extend beyond the nutrient needs of the patient.

## Abbreviations

BA: Bile acid
CREB: cAMP response element-binding protein
FXR: Farnesoid-X-receptor
HDL: High-density lipoprotein
IL: Interleukin
LPS: Lipopolysaccharide
NO: Nitric oxide
PPARα: Peroxisome proliferator–activated receptor-alpha
SAA: Sickness-associated anorexia
SR-BI: Scavenger receptor class B type I
TGR5: Takeda G protein–coupled receptor 5
TNF: Tumour necrosis factor
